# Thymectomy in ocular myasthenia gravis—prognosis and risk factors analysis

**DOI:** 10.1186/s13023-022-02454-y

**Published:** 2022-08-09

**Authors:** Jinwei Zhang, Zeyang Zhang, Hui Zhang, Yuantao Cui, Yuan Chen, Peng Lv, Peng Zhang

**Affiliations:** Department of Cardiothoracic Surgery, Tianjin Medical University General Hospital, No.154, Anshan Road, Tianjin, China

**Keywords:** Conversion, Ocular myasthenia gravis, Remission, Thymectomy, Thymoma

## Abstract

**Background:**

Several retrospective studies have identified risk factors associated with ocular myasthenia gravis (OMG) generalization in non-surgical patients. However, the outcomes of OMG after thymectomy have not been investigated fully. This study aimed to explore the clinical predictors of post-thymectomy OMG prognosis.

**Methods:**

We performed a retrospective review of OMG patients who underwent thymectomy at our institution from January 2012 to December 2021. Kaplan–Meier and Cox proportional hazard regression analyses were used to evaluate associations between clinical features and prognosis. The main outcome measures were OMG conversion, complete stable remission (CSR), and clinical improvement.

**Results:**

Fifty-eight patients were identified for conversion analysis. Thirteen (22.4%) developed generalized myasthenia gravis (GMG) at a median time of 12.7 (3–37.3) months from symptom onset. Repetitive nerve stimulation (RNS)-positivity was associated with increased risk of conversion to GMG (*P* = 0.002). Patients with histotype B2/B3 thymoma showed a higher risk of conversion (*P* = 0.002) than did patients with hyperplasia and AB/B1 thymoma. Fifty-two patients fulfilled the criteria for CSR and improvement. Sixteen (30.8%) achieved CSR at a median time of 28.7 (15–54) months after thymectomy. Fifteen (28.8%) showed clinical improvement at last follow up. Patients who achieved CSR showed a younger age of onset (*P* = 0.022), lower percentage of acetylcholine receptor antibody-seropositivity (*P* = 0.029). Histologically, patients with thymic hyperplasia and stage I thymoma showed a higher chance of CSR (*P* = 0.010) than did patients with stage II/III thymoma. Multivariate analysis revealed that RNS-positivity (hazard ratio [HR] 6.007, *P* = 0.021) and histotype B2/B3 thymoma (HR 4.611, *P* = 0.048) were associated with OMG conversion. Thymic hyperplasia and stage I thymoma (HR 0.300, *P* = 0.026) were associated with OMG CSR after thymectomy.

**Conclusion:**

For OMG patients after thymectomy, RNS-positivity and histotype B2/B3 thymoma are independent predictors of conversion to GMG. On the other hand, thymic hyperplasia and stage I thymoma independently predict CSR.

## Background

Myasthenia gravis (MG) is an autoimmune disease caused by pathogenic autoantibodies to components of the postsynaptic muscle endplate. The typical manifestation is fluctuations in severity of muscle weakness [1]. According to symptoms at disease onset, MG can be further divided into ocular MG (OMG, MGFA class I) and generalized MG (GMG, MGFA Class II–V) [2]. However, 50–65% of OMG patients will develop systemic neuromuscular weakness, indicating conversion to secondary GMG (SGMG), typically within the first 2 years [3].

Patients with pure OMG and SGMG can differ significantly in the clinical course and impairment of daily life. Several retrospective studies have identified certain risk factors associated with OMG conversion, which include sex, older age of onset [4], antibody-seropositivity [5], and the presence of other autoimmune diseases [6]. However, most of these studies recruited patients treated conservatively (with pyridostigmine or immunosuppressants), and data on OMG prognosis after thymectomy are rare. Moreover, the thymus is pathologically linked to MG. A significant correlation of thymoma histologic subtype, Masaoka stage, and MG pathogenesis has been described by Weis et al. [7]. They found that the immunopathology of types B1–B3 thymomas favour lack of self-tolerance and triggering of MG [7]. Whether these differences would translate to differences in the post-thymectomy outcomes of OMG is intriguing. Furthermore, thymomas can vary in size and location in the anterior mediastinum, but it is unknown whether these anatomical features could impact thymoma-associated OMG prognosis [8].

In view of the rarity of studies on surgery in OMG, this study aimed to address two issues pertinent to OMG patients post-thymectomy: we sought to report on long-term post-thymectomy OMG prognosis and to explore factors affecting post-thymectomy OMG outcomes.

## Materials and methods

### Patient enrolment and definition

We conducted a retrospective study of 82 consecutive OMG patients who underwent thymectomy at Tianjin Medical University General Hospital from January 2012 to December 2021. The diagnosis of OMG was based on typical clinical manifestations, consisting of fluctuating diplopia, ptosis, or both, and at least one positive result in the following tests: (1) Anti-AChR Ab, (2) RNS, or (3) clinical response to edrophonium chloride (Tensilon test) or pyridostigmine.

Outcome measures consisted of development of GMG, complete stable remission (CSR) and clinical improvement, time to OMG conversion (calculated from time of symptom onset) and to CSR (calculated from thymectomy).

Inclusion criteria were an onset age ≥ 18 years, a minimum of 3 months of isolated ocular disease, R0 resection for thymoma, and follow-up duration of at least 2 years: 2 years or more from symptom onset or until GMG developed in conversion analysis, and 2 years or more after thymectomy for CSR and clinical improvement analysis. Patients who were lost to follow-up or who had incomplete clinical data were excluded from this study. We also excluded patients with thymic carcinomas or cysts because these conditions have not been validated to be associated with MG pathogenesis [9].

GMG was defined by any symptoms beyond the extraocular muscles or eyelid, including dysphagia, dysarthria, dyspnoea, dysphonia, neck or extremity weakness, with the above positive serological or physiological testing. CSR was defined as the absence of any symptoms or signs of MG for at least 1 year without any medication for MG. Clinical improvement was defined as a substantial decrease in pre-treatment clinical manifestations or a sustained substantial reduction in MG medications.

Extended thymectomy, defined as resection of the entire thymus and mediastinal fat tissue between both phrenic nerves, was performed for all patients. Indications for thymectomy included thymic abnormality suggestive of thymoma on diagnostic imaging, or in suspicion of hyperplasia but with inadequate response to acetylcholinesterases, resistance to taking immunosuppressive (IS) therapy or with contraindications to or being refractory to IS agents. With respect to OMG patients in whom it was difficult to differentiate thymoma from thymic hyperplasia in thymic imaging, we still recommended thymectomy, to eliminate any possible thymoma. For patients with a high suspicion of thymic hyperplasia, it was recommended that they make their own decision regarding surgery, after a comprehensive explanation of the pros and cons of both medical treatment and surgery.

This study was approved by the ethics committee of Tianjin Medical University General Hospital (Ethical No. IRB2022-WZ-024) and was conducted according to the principles of the Declaration of Helsinki. The need for obtaining patient consent was waived due to the nature of the study.

**Fig. 1:**
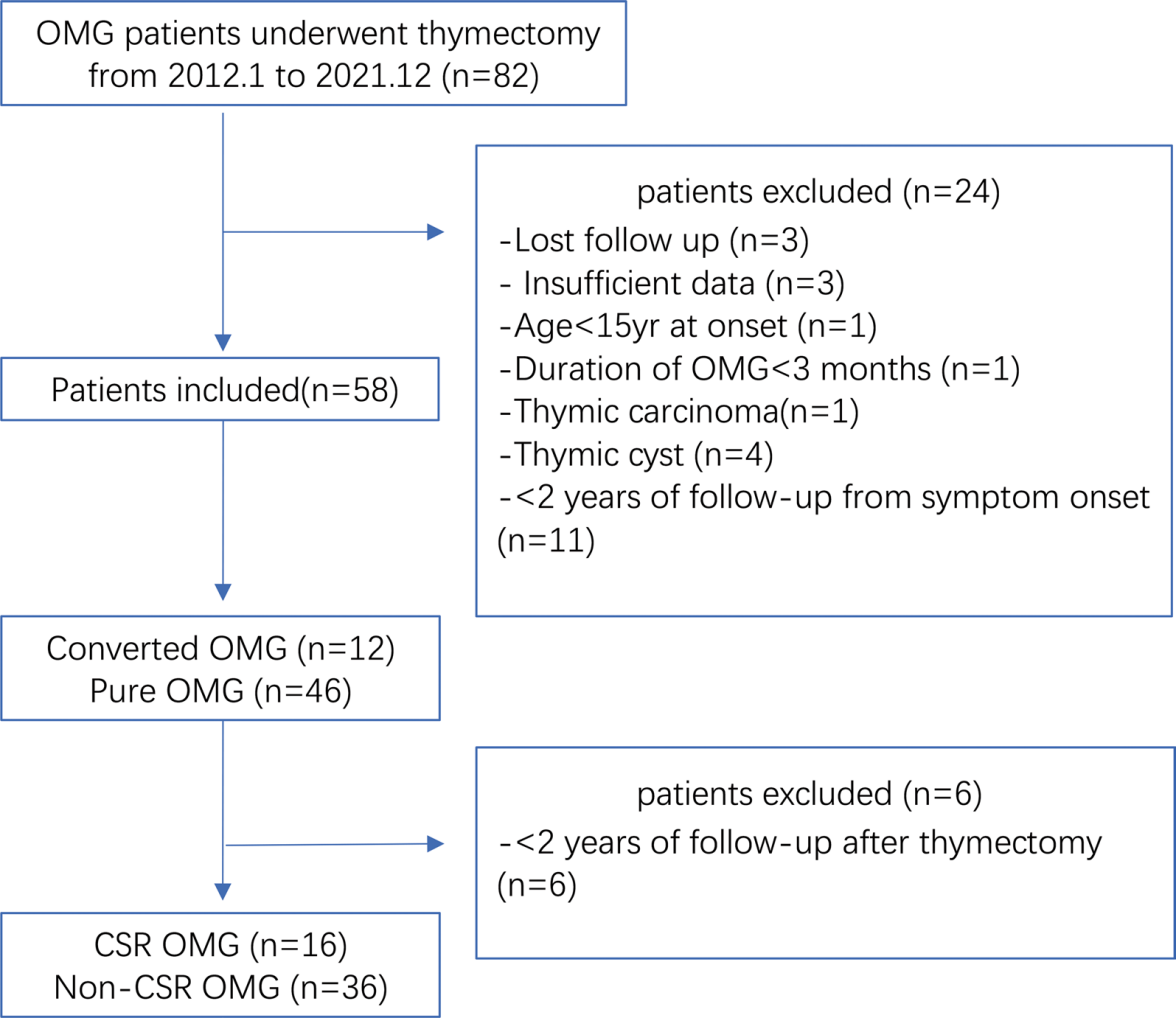
Flowchart of recruitment and exclusion process of OMG patients after thymectomy. OMG, ocular myasthenia gravis. CSR, complete stable remission

After inclusion and exclusion criteria screening, twenty-four patients were excluded from the study. Therefore, a total of 58 patients were finally identified for OMG conversion study, 52 patients were eligible for CSR analysis. The flowchart of recruitment and exclusion detail was shown in Fig. 1.

### Clinical predictors

The following variables that may predict conversion and CSR were evaluated: age, sex, clinical symptoms at onset (diplopia, ptosis, or both), anti-AChR Ab statis, RNS status, thymus histology, Masaoka–Koga Stage of thymoma, tumour location, tumour size, surgical approach, duration of symptoms before surgery, and immunosuppressive treatment (corticosteroids, azathioprine or tacrolimus) after surgery.

### Thymus pathology

Thymoma histology was classified according to the WHO criteria [10] by local surgical pathologists. To facilitate analysis, we assigned each thymoma to one of the WHO subtypes (A, AB, B1, B2, B3). However, six cases had combinations of type B1 and B2, and five cases had combinations of type B2 and B3 thymomas. We classified these “B1 plus B2” as B2 thymomas, and “B2 plus B3” as B3 thymomas, when there was any area in which the diagnostic histology of B2 or B3 could be recognized. The classification system of Masaoka–Koga [11] was adopted as the staging system. The stage was determined by reviewing the surgical records and pathologic reports.

### Thymoma anatomical features

The tumour location and size were determined from preoperative thoracic imaging examinations. If the tumour boundary exceeded the left/right sternal border, it was defined as left or right thymoma, respectively. Both maximum and mean tumour diameter (measured as the average diameter of the anteroposterior, vertical, and transverse span length) were evaluated.

### Serological testing

Both AchR and MuSK Abs were tested in all patients. The presence of AChR Abs was considered positive with the titre > 0.5 nmol/L, and the presence of MuSK Ab was considered when the titre > 0.01 nmol/L, as assessed in radioimmune assays.

### Electrodiagnostic testing

The RNS test was routinely conducted in the bilateral orbicularis oculi muscle for OMG. A decrement of more than 10% with 3-Hz stimulation was considered to represent a positive result.

### Post-thymectomy treatment

Pyridostigmine alone or in combination with prednisone were prescribed by neurologists, according to OMG symptoms before surgery Generally, prednisone was recommended for patients with diplopia. After symptomatic control was achieved, the dose was tapered over months to the minimum effective dose or withdrawal. When corticosteroids were ineffective, or when side effects limited their use, or when contraindications precluded their use entirely, additional immunosuppressive agents, such as tacrolimus, azathioprine, mycophenolate mofetil, or methotrexate were considered.

### Statistical analysis

Categorical variables were analysed using the chi-square and Fisher’s exact test. Continuous variables were analysed with two-tailed *t-*tests. Cumulative incidence of OMG conversion and CSR were analysed using the Kaplan–Meier method and the log-rank test. Univariate and multivariate Cox proportional hazards regression analysis was applied to determine the factors affecting OMG conversion and CSR during the follow-up period. A P value < 0.05 was considered statistically significant. All data analyses were performed using SPSS Statistics for Mac version 25.0 (IBM Corp, Armonk, NY).

## Results

### Baseline demographics and clinical features

#### Clinical features of converted OMG and pure OMG

The median follow-up duration after thymectomy in our 58 OMG patients was 59.3 (range 9–114.5) months. At last review, 13 (22.4%) patients had developed GMG at a median conversion time of 9.2 (range 1.4–32.9) months after thymectomy, and 12.7 (range 3–37.3) months after symptom onset. Eleven of these 13 patients (84.6%) developed GMG within 2 years of symptom onset. Of the 58 included patients, 24 (42.3%) were men and 34 (57.7%) were women, with a median age at symptom onset of 54.7 years. The clinical characteristics of the 13 OMG patients who converted to GMG and the 45 pure OMG patients are displayed in Table 1. No significant difference was observed in terms of sex, age, OMG symptoms at onset (ptosis/diplopia), disease duration before surgery, surgical approach, tumour location, tumour size, immunosuppressive treatment after surgery, and post-operative follow-up duration. Acetylcholine receptor antibody (AchR Ab)-seropositivity showed a trend for increased risk of conversion to GMG (*P* = 0.085), although no statistically significant difference was reached. RNS-positivity was associated with an increased risk of conversion to GMG, with 84.6% of those with RNS-positivity converting to GMG, as compared with 35.6% of patients who were RNS-negative (*P* = 0.002). We did not find a difference in conversion rate between OMG with thymoma and hyperplasia (12/44, 27.3% vs. 1/14, 7.1%; *P* = 0.228, Table 1). However, patients with histological subtype B2/B3 thymoma showed a statistically significant increase in the risk of conversion (*P* = 0.002) compared to the group of patients with either hyperplasia or subtype AB/B1 thymoma.

**Table 1:** Clinical characteristics of converted OMG and pure OMG

	Overall(58)	Converted OMG (13)	Pure OMG (45)	*p* value
Gender, n (%)				0.300
Male	24 (41.4%)	7 (53.8%)	17 (37.8%)	
Female	34 (58.6%)	6 (46.2%)	29 (62.2%)	
Onset age (years), mean ± SD	55.1 ± 13.2	56.3 ± 10.6	54.4 ± 14.1	0.646
Ptosis, n (%)				0.448^b^
Left	19 (32.8%)	4 (30.8%)	15 (33.3%)	
Right	16 (27.6%)	2 (15.4%)	14 (31.1%)	
Bilateral	23 (39.7%)	7 (53.8%)	17 (35.6%)	
Diplopia ( +), n (%)	25 (43.1%)	7 (53.8%)	18 (40.0%)	0.375
Anti-AchR Ab ( +), n (%)	40 (69.0%)	12 (92.3%)	28 (62.2%)	0.085
RNS ( +), n (%)	27 (46.6%)	11 (84.6%)	16 (35.6%)	0.002*
Disease duration before surgery (weeks), mean ± SD	19.3 ± 44.4	15.0 ± 27.1	20.5 ± 48.5	0.699
Surgical approach, n (%)				0.562^b^
R/L-VATS	22 (37.9%)	4 (30.8%)	18 (40.0%)	
TS	8 (13.8%)	3 (23.1%)	5 (11.1%)	
Sub-xiphoid	28 (48.3%)	6 (46.2%)	22 (48.9%)	
Tumor location, n (%)				0.624^b^
Left	23 (52.3%)	7 (58.3%)	16 (50.0%)	
Right	19 (43.2%)	4 (33.3%)	15 (46.9%)	
L + R^a^	2 (4.5%)	1 (8.3%)	1 (3.1%)	
Maximum diameter (cm), mean ± SD	3.8 ± 1.6	3.5 ± 1.3	3.9 ± 1.7	0.441
Mean size (cm), mean ± SD	3.1 ± 1.2	2.8 ± 1.0	3.2 ± 1.3	0.351
Thymoma histotype + hyperplasia, n (%)				0.137^b^
Thymoma				
AB	10 (17.2%)	1 (7.7%)	9 (20.0%)	
B1	4 (6.9%)	0	4 (8.9%)	
B2	20 (34.5%)	7 (53.8%)	13 (28.9%)	
B3	10 (17.2%)	4 (30.8%)	6 (13.3%)	
Hyperplasia	14 (24.1%)	1 (7.7%)	13 (28.9%)	
Thymoma: Hyperplasia	44:14	12:1	32:13	0.228
B2 + B3: AB + B1 + hyperplasia	30:28	11:2	19:26	0.007*
Thymoma Stage + hyperplasia, n (%)				0.095^b^
Thymoma				
I	11 (19.0%)	4 (30.8%)	7 (15.6%)	
IIa	12 (20.7%)	1 (7.7%)	11 (24.4%)	
IIb	13 (22.4%)	3 (23.1%)	10 (22.2%)	
III	8 (13.8%)	4 (30.8%)	4 (8.9%)	
Hyperplasia	14 (24.1%)	1 (7.7%)	13 (28.9%)	
NTOMG, n (%)				1.000^b^
Early-onset (age ≤ 50)	7 (12.1%)	0	7 (15.6%)	
Late-onset (age > 50)	7 (12.1%)	1 (7.7%)	6 (13.3%)	
Post-operative prednisone ( +), n (%)	20 (34.5%)	4 (30.8%)	16 (35.6%)	1.000
Post-operative follow-up duration (months), mean ± SD	59.3 ± 30.3	55.5 ± 36.3	60.5 ± 28.8	0.608

Values in boldface as well as * indicate statistically significant

*OMG* ocular myasthenia gravis, *NTOMG* non-thymomatous OMG, *AChR-ab* anti-acetylcholine receptor antibody, *RNS* repetitive nerve stimulation, *R/L-VATS* right/left-video assisted thoracoscopic surgery, *TS* trans-sternal

^*^Statistically significant

^a^Tumor boundary exceeds both sides of sternal border

^b^Fisher exact test

#### Clinical features of CSR-OMG and non-CSR OMG

Of the 52 patients who could be included in the CSR analysis, 16 (30.8%) achieved CSR at a median time of 28.7 (range 15–54) months after thymectomy. Fifteen (28.8%) patients showed improvement at the last follow-up. No significant difference was observed between the CSR-OMG and non-CSR OMG patients in terms of sex, OMG symptoms at onset, disease duration before surgery, surgical approach, tumour location, tumour size, or immunosuppressive treatment after surgery. However, patients who achieved CSR had a younger age of onset (48.3 ± 13.7 vs. 57.5 ± 12.6 years, *P* = 0.022).

**Fig. 2:**
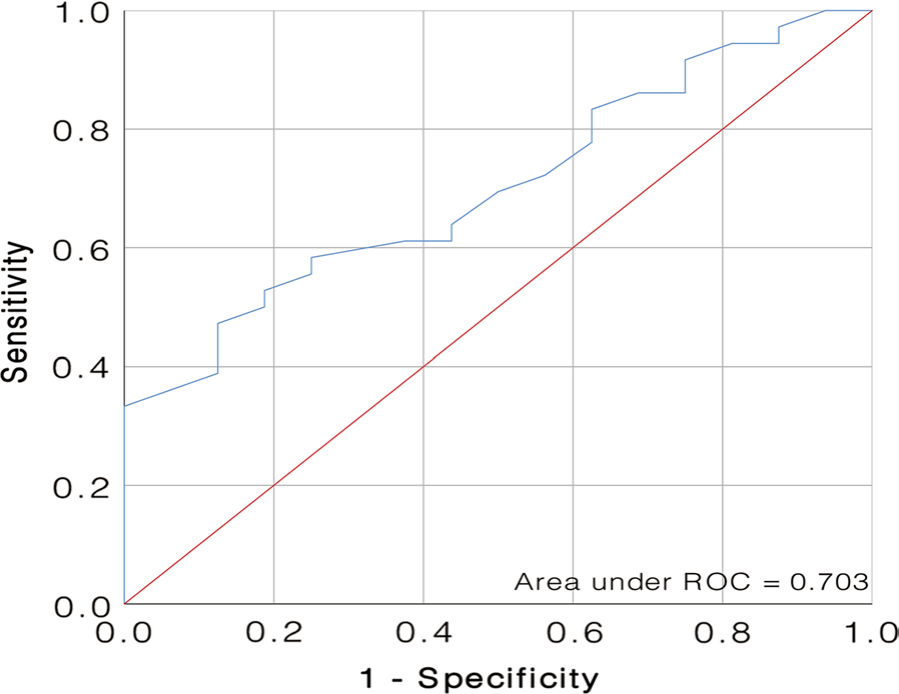
Estimated probability of CSR and ROC curve of onset age (60.5 years, AUC = 0.703, 95% CI 0.561–0.846, sensitivity: 47.2%, specificity: 87.5%). CSR, complete stable remission. ROC, receiver operating characteristic. AUC, area under the ROC curve. CI, confidence interval

We then used receiver operating characteristic (ROC) curves to explore the best threshold for age (60.5 years, area under the ROC curve [AUC] 0.703, 95% confidence interval [CI] 0.561–0.846; sensitivity: 47.2%; specificity: 87.5%) (Fig. 2). The AchR Ab-seropositivity was associated with a decreased probability of CSR (*P* = 0.029). RNS-positivity was associated with a trend for decreased probability of CSR (*P* = 0.063). Patients with thymic hyperplasia or stage I thymoma showed a significantly higher chance of achieving CSR (*P* = 0.010) compared with patients with stage II/III thymoma. Post-operative follow-up duration was markedly longer in the CSR group (78.3 ± 28.6 vs. 58.3 ± 25.4 months, *P* = 0.015), indicating that, with the extension of follow-up time, more patients would achieve CSR and that 2 years may not be long enough to determine whether CSR can be reached. We believe that this follow-up duration difference also explained the surgical approach outcomes between these two groups, as unilateral video-assisted thoracoscopic surgery was routinely performed from 2012 to 2017 in our centre, while the sub-xiphoid method became mainstream in the recent 4 years (Table 2).

**Table 2:** Clinical characteristics of CSR OMG and non-CSR OMG

	Overall (52)	CSR OMG (16)	Non-CSR OMG (36)	*p* value
Gender, n (%)				0.282
Male	22 (42.3%)	5 (31.3%)	17 (47.2%)	
Female	30 (57.7%)	11 (68.8%)	19 (52.8%)	
Onset age (years), mean ± SD	54.7 ± 13.5	48.3 ± 13.7	57.5 ± 12.6	0.022*
Onset age (years), n (%)				0.016*
≤ 60	33 (63.5%)	14 (87.5%)	19 (52.8%)	
> 60	19 (36.5%)	2 (12.5%)	17 (47.2%)	
Ptosis, n (%)				0.765^c^
Left	18 (34.6%)	6 (37.5%)	12 (33.3%)	
Right	14 (26.9%)	5 (31.3%)	9 (25.0%)	
Bilateral	20 (38.5%)	5 (31.3%)	15 (41.7%)	
Diplopia ( +), n (%)	21 (40.4%)	9 (56.3%)	12 (33.3%)	0.120
Anti-AchR Ab ( +), n (%)	34 (65.4%)	7 (43.8%)	27 (75.0%)	0.029*
RNS ( +), n (%)	23 (44.2%)	4 (25.0%)	19 (52.8%)	0.063
Disease duration before surgery (weeks), mean ± SD	19.4 ± 46.6	8.6 ± 9.9	24.1 ± 55.2	0.111
Surgical approach, n (%)				0.056^c^
R/L-VATS	22 (42.3%)	10 (62.5%)	12 (33.3%)	
TS	8 (15.4%)	3 (18.8%)	5 (13.9%)	
Sub-xiphoid	22 (42.3%)	3 (18.8%)	19 (52.8%)	
Tumor location, n (%)				0.488^b^
Left	19 (48.7%)	4 (40.0%)	15 (51.7%)	
Right	18 (46.2%)	5 (50.0%)	13 (44.8%)	
L + R^a^	2 (5.1%)	1 (10%)	1 (3.4%)	
Maximum diameter (cm) mean ± SD	3.8 ± 1.6	3.6 ± 2.2	3.9 ± 1.4	0.592
Median size (cm) mean ± SD	3.1 ± 1.3	3.0 ± 1.7	3.1 ± 1.1	0.739
Thymoma histotype + hyperplasia, n (%)				0.330^b^
Thymoma				
AB	9 (17.3%)	1 (6.3%)	8 (22.2%)	
B1	4 (7.7%)	2 (12.5%)	2 (5.6%)	
B2	18 (34.6%)	4 (25.0%)	14 (38.9%)	
B3	8 (15.4%)	3 (18.8%)	5 (13.9%)	
Hyperplasia	13 (25.0%)	6 (37.5%)	7 (19.4%)	
Thymoma Stage + hyperplasia, n (%)				0.086^b^
Thymoma				
I	9 (17.3%)	5 (31.3%)	4 (11.1%)	
IIa	12 (23.1%)	1 (6.3%)	11 (30.6%)	
IIb	12 (23.1%)	2 (12.5%)	10 (27.8%)	
III	6 (11.5%)	2 (12.5%)	4 (11.1%)	
Hyperplasia	13 (25.0%)	6 (37.5%)	7 (19.4%)	
Hyperplasia: thymoma	13:39	6:10	7:29	0.298
Hyperplasia + I: II + III	22:30	11:5	11:25	0.010*
NTOMG, n (%)				0.592^b^
Early-onset (age ≤ 50)	7 (13.5%)	4 (25%)	3 (8.3%)	
Late-onset (age > 50)	6 (11.5%)	2 (12.5%)	4 (11.1%)	
Post-operative prednisone ( +), n (%)	18 (34.6%)	5 (31.3%)	13 (36.1%)	0.734
Post-operative follow-up duration (months), mean ± SD	60.5 ± 27.7	78.3 ± 28.6	58.3 ± 25.4	0.015*

Values in boldface as well as * indicate statistically significant

*CSR* complete stable remission, *OMG* ocular myasthenia gravis, *NTOMG* non-thymomatous OMG, *AChR-ab* anti-acetylcholine receptor antibody, *RNS* repetitive nerve stimulation, *R/L-VATS* right/left-video assisted thoracic surgery, *TS* trans-sternal

^*^Statistically significant

^a^Tumor boundary exceeds both sides of sternal border

^b^Fisher exact test

^c^Likelihood Ratio test

### Cumulative probability of OMG conversion and CSR

**Fig. 3:**
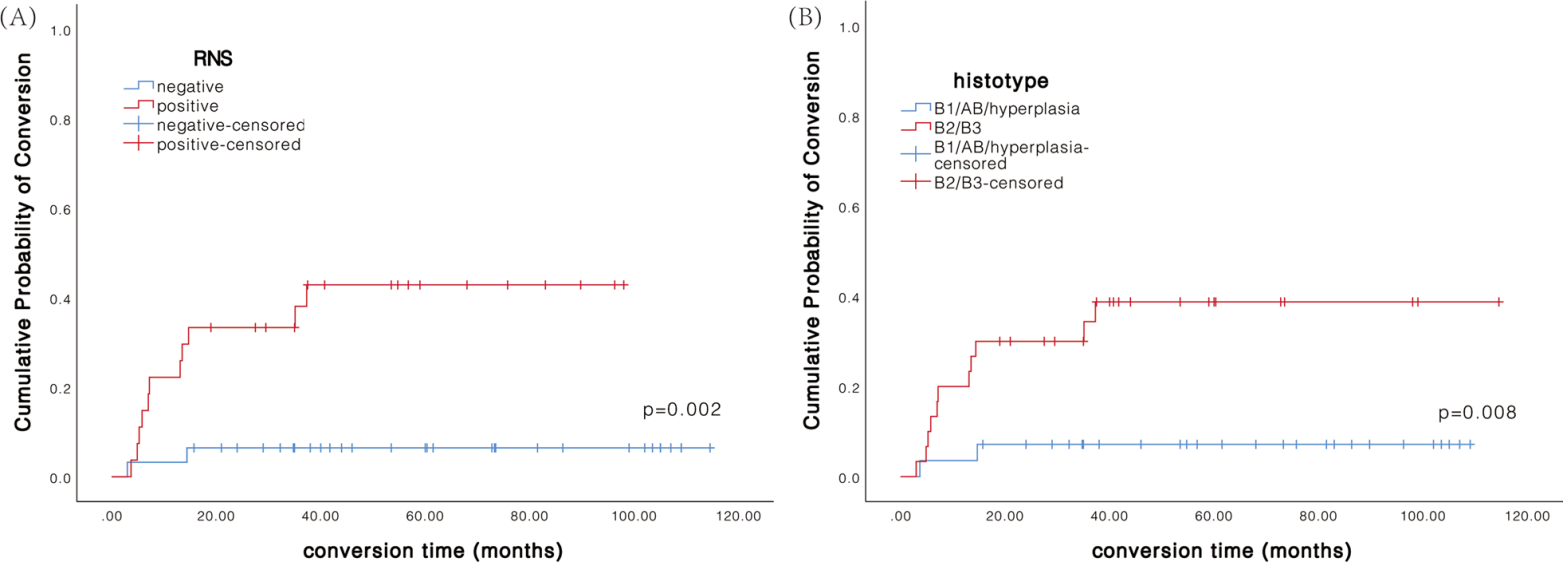
Kaplan–Meier curve of the cumulative probability of conversion to GMG after onset of symptoms in in different patient groups. **A** Patients with negative and positive RNS results (*P* = 0.002). **B** patients with different thymic histology (*P* = 0.008). GMG, generalized myasthenia gravis. RNS, repetitive nerve stimulation

The cumulative probability of OMG conversion in the sample of 58 patients, according to RNS, AchR Ab status, and thymus histology (B2/B3 versus hyperplasia + AB/B1), was calculated by using the Kaplan–Meier method. Among these variables, RNS-positivity (*P* = 0.002) and histological subtype of B2/B3 thymoma (*P* = 0.008) were associated with increased risk of OMG conversion as compared with patients with RNS-negativity and histotype hyperplasia + AB/B1 thymoma (Fig. 3).

**Fig. 4:**
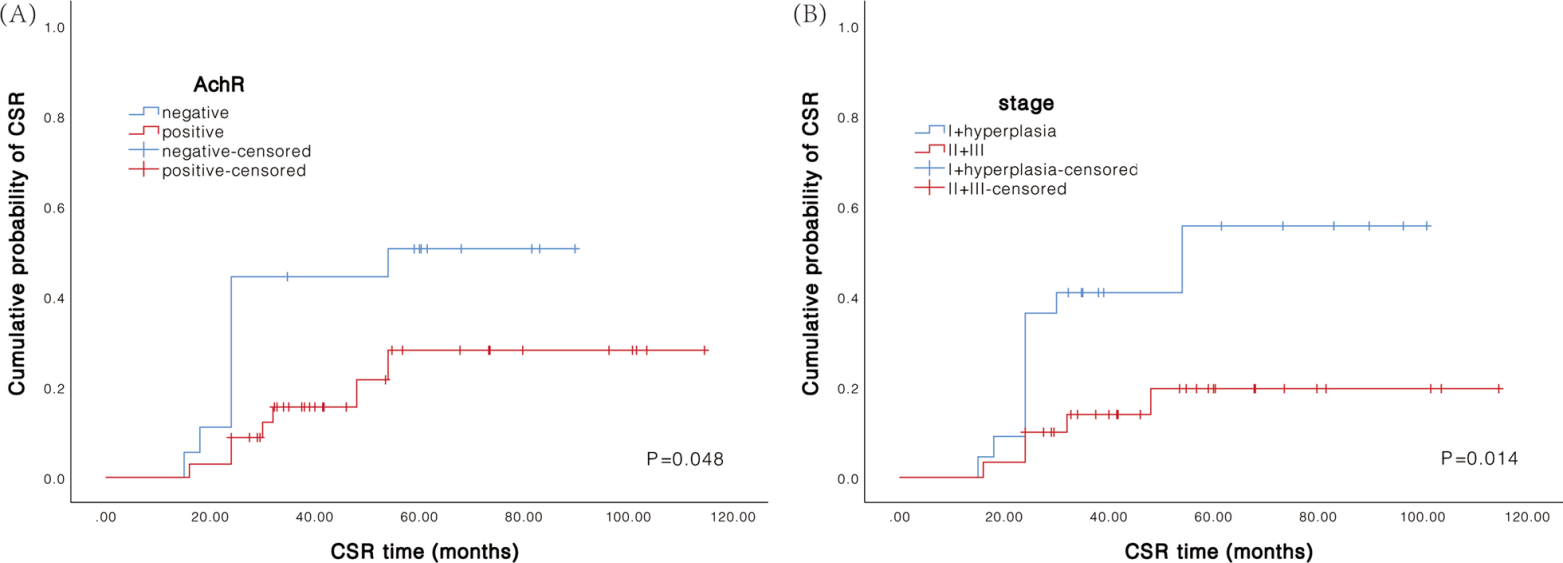
Kaplan–Meier curve of the cumulative probability of CSR after thymectomy in different patient groups. **A** Patients with negative and positive anti-AchR Ab (*P* = 0.048). **B** Patients with different thymic stages (*P* = 0.014). CSR, complete stable remission. AchR Ab, acetylcholine receptor antibody

The cumulative probability of achieving CSR was also studied. The analysis showed that AchR Ab-seronegativity (*P* = 0.048) was associated with a higher chance of achieving CSR. Patients with hyperplasia and stage I thymoma showed an increased chance of achieving CSR as compared to those with stage II–III thymoma (*P* = 0.014) (Fig. 4).

### Multivariate analysis

Both univariate and multivariate analysis, using a Cox proportional hazard model, were performed to verify prognostic factors associated with OMG conversion and CSR. Among the variables considered, RNS-positivity (hazard ratio [HR] 6.007, 95% CI 1.316–27.412, *P* = 0.021) and histotype B2/B3 thymomas (HR 4.611, 95% CI 1.010–21.043, *P* = 0.048) were significantly associated with OMG conversion. On the other hand, thymic hyperplasia and Masaoka–Koga Stage I thymoma (HR 0.300, 95% CI 0.104–0.864, *P* = 0.026) were independently associated with CSR in OMG patients after thymectomy (Table 3). We further performed subgroup analysis for patients with thymomatous OMG, and found by univariable Cox regression analysis that RNS positivity was a significant positive predictor for OMG conversion (HR 5.274, 95% CI 1.54–24.106, *P* = 0.032), and an age at onset > 60 years was a significant negative predictor for achieving CSR (HR 0.124, 95% CI 0.016–0.981, *P* = 0.048). In multivariate Cox analysis, only RNS positivity was a significant positive predictor for OMG conversion (HR 5.017, 95% CI 1.094–22.997, *P* = 0.038). Thymoma histotype B2/B3 showed a positive association with OMG conversion compared to AB/B1, and stage II/III thymoma showed a negative association with CSR compared to the stage I counterpart. However, neither reached statistical significance (Table 4).

**Table 3:** Univariate and multivariate Cox regression analysis for OMG conversion and CSR

	Univariate		Multivariate	
	HR (95% CI)	*p* value	HR (95% CI)	*p* value
Conversion				
RNS (+)	7.462 (1.651–33.718)	0.009*	6.007 (1.316–27.412)	0.021*
Anti-AchR Ab (+)	6.428 (0.832–49.681)	0.075		
B2/B3 thymoma versus Hyperplasia + AB/B1 thymoma	5.987 (1.325–27.044)	0.020*	4.611 (1.010–21.043)	0.048*
CSR				
Onset age > 60 versus ≤ 60	0.230 (0.052–1.011)	0.052		
RNS (+)	0.378 (0.122–1.175)	0.093		
Anti-AchR Ab (+)	0.396 (0.147–1.068)	0.067		
Stage II/III thymoma versus Hyperplasia + stage I thymoma	0.300 (0.104–0.864)	0.026*	0.300 (0.104–0.864)	0.026*

Values in boldface as well as * indicate statistically significant

*CSR* complete stable remission, *OMG* ocular myasthenia gravis, *AChR-ab* anti-acetylcholine receptor antibody, *RNS* repetitive nerve stimulation, *HR* hazard ratio

^*^Statistically significant

**Table 4:** Univariate and multivariate Cox regression analysis for OMG conversion and CSR in thymomatous subtype

	Univariate		Multivariate	
	HR (95% CI)	*p* value	HR (95% CI)	*p* value
Conversion				
RNS (+)	5.274 (1.154–24.106)	0.032*	5.017 (1.094–22.997)	0.038*
B2/B3 versus AB/B1	6.158 (0.794–47.738)	0.082	5.771 (0.742–44.872)	0.094
CSR				
Onset age > 60 versus ≤ 60	0.124 (0.016–0.981)	0.048*	0.138 (0.017–1.091)	0.060
Stage II/III versus I	0.298 (0.086–1.031)	0.056	0.298 (0.099–1.202)	0.095

Values in boldface as well as * indicate statistically significant

*CSR* complete stable remission, *OMG* ocular myasthenia gravis, *RNS* repetitive nerve stimulation, *HR* hazard ratio

^*^Statistically significant

## Discussion

To the best of our knowledge, no previous study had simultaneously targeted predictors of GMG conversion and CSR after thymectomy in OMG patients. Our retrospective study showed that RNS-positivity and World Health Organization (WHO) subtype B2/B3 thymomas were significantly associated with post-thymectomy OMG conversion. On the other hand, thymic hyperplasia and Masaoka–Koga Stage I thymoma were independently associated with post-thymectomy OMG CSR.

Thymectomy is required for patients with thymoma. Its role in non-thymomatous OMG (NTOMG), however, remains highly controversial [12]. In our department, patients with OMG underwent thymectomy on condition of thymus abnormalities found on diagnostic imaging. Fourteen patients with pathologically confirmed thymic hyperplasia were included in our study. Thymectomy has already been shown to result in better clinical outcomes and reduced corticosteroid requirements in non-thymomatous AchR-seropositive GMG [13]. The same potential benefits may also apply in OMG patients, particularly in those with thymic hyperplasia. Correlations between the degree of follicular hyperplasia and the level of anti-AChR Abs suggest a causal relationship between thymic hyperplasia and MG [14]. Wong et al. [6] identified that thymic hyperplasia was a significant predictive factor for conversion to GMG in OMG patients who have not undergone thymectomy. Wang et al. [15] reported that OMG patients with thymus hyperplasia progressed more rapidly than did those with other thymus pathology. Patients with thymic hyperplasia in our study cohort showed a satisfying post-thymectomy outcome (only 1 of 14 patients converted to GMG, whereas 6 of 13 achieved CSR), which was in accord with some other previous studies [16, 17]. Histologically, MG was reportedly associated mainly with type B thymomas and tended to be more frequent in type B2 or B3 thymomas [7]. Our study supported the correlation between thymus pathology and OMG prognosis.

Although no correlation was detected between OMG generalization and AChR Ab status, our study showed that patients who were seronegative for AChR Abs were more likely to achieve CSR after thymectomy. Using standard assays, AChR Abs are detectable in approximately 50% of patients with OMG and in nearly 90% of patients with GMG [2]. Anti-muscle-specific receptor tyrosine kinase (MuSK) Abs are rarely found in isolated OMG [18]. Only one patient who was AChR Ab-seronegative was anti-MuSK Ab-seropositive in our study. We did not incorporate this variable into our analysis because of this small proportion. However, one must be aware that many “seronegative” cases may harbour an autoantibody that is not detected by conventional means. The diagnostic yield of AChR Ab testing is significantly increased by cell-based assays [19], rather than by the typical radioimmunoprecipitation technique. Moreover, other autoantibodies may also exist. Anti-LRP4 Abs have been detected in about 20% of “double-negative” MG [20]. Antibodies directed against cortactin (a postsynaptic protein required for clustering of AChRs) have been identified in 24% of double-seronegative OMG patients [21]. Whether the presence of these antibodies could have an effect on OMG prognosis requires further validation.

The presence of comorbidities at disease onset has been described as a risk factor for conversion to GMG [6]. We did not incorporate this variable into analysis because of the small sample size (only 6 of 58 patients had another comorbid autoimmune disease). This link will need to be further validated externally for more robust evidence.

At our institution, we consider a minimum of 3 months of isolated ocular disease as an inclusion criterion. This 3-month duration is in keeping with the views of other studies [6]. Monsul et al. [22] suggested this time interval as the limit for purely ocular symptoms before classifying a patient as having OMG. However, studies by Kupersmith [23] and Mee et al. [24] included patients who developed GMG within 3 months of symptom onset. This is an important question that should be clarified in future studies.

Our analyses did not identify age as a risk factor for OMG conversion. The effect of age on OMG conversion to GMG is paradoxical among studies. Feng et al. [4] revealed that the onset age correlated with the conversion rate, and the threshold age was 43 years. On the other hand, Wong et al. did not show age as a risk factor in either univariable or multivariable analysis [6]. However, we did find that younger patients showed a greater chance of achieving CSR than did the older patients. ROC curve analysis indicated that the best threshold for age was 60.5 years (Fig. 2), which is older than the usual age limit for distinguishing early from late onset MG [2]. We attributed this cut-off age discrepancy to demographic characteristic differences. Because most patients in our study had thymoma-related OMG, 50 years may not be suitable as a cut-off age in our study cohort, or in an OMG with thymoma cohort. Moreover, Sarkkinen et al. [25] recently reported that ectopic germinal centres, which can predict better post-thymectomy outcomes in NTMG, are more prevalent in early onset NTMG thymus. In consideration of the potential differences in thymic histopathology in NTMG patients of different ages, we further analysed the proportion of early-onset and late-onset cases in our NTOMG sample set. Early onset cases accounted for almost the same proportion as late onset counterparts, both in terms of conversion and of CSR analysis (Tables 1 and 2). For OMG patients with hyperplasia, it remains unclear whether age can affect post-thymectomy outcomes; this needs further investigation.

Our study showed that RNS-positivity was strongly associated with OMG conversion in univariable analysis (*P* = 0.002), log-rank test (*P* = 0.002), and multi-variable COX regression analysis (*P* = 0.022). Moreover, RNS-negativity tended to be associated with a greater likelihood of CSR, although the difference did not reach statistical significance. The association between RNS and OMG conversion was reported by other studies [26]. Kim. et al. [27] revealed that an abnormal RNS test, particularly in the limb muscles, was an independent predictor of the conversion from OMG to GMG. In addition, an abnormal RNS test has been reported to be related to a shortened time to conversion to GMG [28]. Since limb muscles were not routinely tested for OMG in our study, whether performing RNS testing in orbicularis oculi would be sufficient for evaluating neurotransmission in extraocular muscles needs further evaluation.

Previous studies have reported the effect of prednisone in delaying onset of GMG as well as its sustained benefit in reducing the incidence of GMG and controlling diplopia [22, 23]. Generally, patients with diplopia were recommended to receive prednisone therapy, as in our study cohort. Safety concerns about corticosteroids usage include the development of hypertension, diabetes mellitus, osteoporosis, gastrointestinal disorders, or infectious illness, which are common with the chronic use of moderate to high doses of the drug. The rate of post-operative prednisone usage did not differ among our OMG sample set, both in conversion and CSR analysis. The administration of corticosteroids to eliminate extraocular muscle limitation and diplopia continues to be controversial. Core issues revolve around selection of clear responders and the balance between benefits and adverse events [3].

Limited studies have discussed the importance of tumour size and location in determining clinicopathological features or its relationship to thymoma or MG prognosis. Tian et al. reported that thymomas located in the superior mediastinum were more likely to be associated with disease progression and tumour recurrence than those located in the inferior mediastinum [29]. Okumura et al. [30] showed that tumour size determines both recurrence-free survival and disease-specific survival after surgical treatment for thymoma, with a higher incidence of recurrence in patients with thymoma > 5.0 cm and of death in patients with thymoma > 8.0 cm. None of these studies ever mentioned the correlation of thymoma anatomic features with MG. Our study intended to reveal an association between thymoma location/size with OMG prognosis, although statistical significance was not achieved.

Few studies have specifically studied OMG remission after thymectomy. Liu et al. [16] reviewed 110 OMG patients who underwent extended transsternal thymectomy: 26.4% achieved CSR and 58.2% showed improvement. Surprisingly, no patient converted to GMG during the follow-up period. Unlike in our study cohort, nearly all (95.7%) of their patients were non-thymomatous. The rationale for their high rate of surgery in OMG without thymoma is unclear. Mineo et al. [31] retrospectively reviewed 47 NTOMG patients after thymectomy, of whom 64% achieved stable remission. Robert et al. [32] documented 61 OMG patients treated with thymectomy, who were followed for a mean duration of 9 years, and reported a cure, defined as asymptomatic status without the need for medication, in 51%. Thymomatous OMG accounted for 19.7% of all their cases. The discrepancy of thymus pathology might be the main difference among these studies. This may partly explain the higher remission rate in some of these studies. As stated above, OMG patients with hyperplasia seems to achieve a better outcome than their counterparts with thymoma. Huang et al. [33] reported the highest CSR rate (70.8%) in OMG patients after thymectomy. The histopathology of the thymus, however, was unclear in their study. Overall, the reasons for discrepancy in the OMG remission rate among studies are uncertain and may be complicated. The heterogeneity in demographics, pre-operative symptoms, definition of remission, peri-operative treatment, and thymus pathology may collaborate in contributing to this discrepancy. Among these studies, only Liu et al. [34] explored potential predictors of remission in OMG patients after thymectomy. They found that only age at onset of 40 years or younger could serve as a predictor of OMG remission. However, the onset age of this study cohort was markedly younger (median 40, range 5–79 years). More importantly, only 17.6% of patients had OMG with thymoma, which is a marked difference from our study. Overall, studies on prognosis of thymomatous OMG after thymectomy are scarce, and our study made a significant contribution in this area.

The limitations of this study should be acknowledged. First, the current study had a retrospective design, which may have caused selection bias. Second, the number of patients with post-thymectomy OMG was small. The risk factors identified from this limited sample size may not be generalizable for all patients. Third, given the difference in follow-up duration between groups in our CSR analysis, our inclusion criterion of a follow-up period of 2 years may have caused selection bias, which might have influenced the CSR rate and other associated risk factors. Fourth, only AChR-Ab and MuSK-Ab status was tested in our study. Whether other autoantibodies, such as anti-LRP4 Abs and anti-cortactin Abs would present as confounding factors remains unknown. Finally, considering the different aetiology and pathogenesis of thymoma and thymus hyperplasia, the rationale of combining patients with hyperplasia and specific subtypes of thymoma is debatable.

In conclusion, we revealed the impact of thymus pathology and thymoma anatomical features on prognosis of OMG after thymectomy. We found that RNS-positivity and histotype B2/B3 thymoma were independent predictors of OMG conversion to GMG. On the other hand, thymic hyperplasia and stage I thymoma independently predicted CSR after thymectomy. Patients with a younger age of onset and who are negative for anti-AchR Abs may also have a higher chance of achieving CSR. Further prospective studies with a larger number of patients are warranted to validate our findings.

## Data Availability

All data were retrieved from the medical record database of Tianjin Medical University General Hospital. Please contact the corresponding author for data requests.

## Abbreviations

AchR Ab: Acetylcholine receptor antibody
AUC: Area under the curve
CI: Confidence interval
CSR: Complete stable remission
GMG: Generalized myasthenia gravis
HR: Hazard ratio
MG: Myasthenia gravis
MuSK Ab: Muscle-specific receptor tyrosine kinase antibody
NTOMG: Non-thymomatous ocular myasthenia gravis
OMG: Ocular myasthenia gravis
RNS: Repetitive nerve stimulation
ROC: Receiver operating characteristic
SGMG: Secondary generalized myasthenia gravis
